# Depression symptoms in perimenopausal women with somatic pain: nomogram construction based on a logistic regression model

**DOI:** 10.3389/fpubh.2025.1528718

**Published:** 2025-05-09

**Authors:** Feng Gao, Shinian Zhang, Zenan Sun, Weijian Wang

**Affiliations:** ^1^Affiliated Hospital of Nanjing University of Chinese Medicine, Nanjing, China; ^2^Jiangsu Province Hospital of Chinese Medicine, Nanjing, China; ^3^Management School, Nantong University, Nantong, China

**Keywords:** perimenopausal women, somatic pain, depression, influencing factors, LASSO, logistic regression

## Abstract

**Objective:**

This study investigated the factors influencing depressive symptoms in women with somatic pain during the perimenopausal period in China and established and validated a nomogram prediction model.

**Methods:**

The predictive model is based on data from the China Health and Retirement Longitudinal Study (CHARLS), which focused on individuals aged 45–59 years with somatic pain during the perimenopausal period. The study utilized participants from the CHARLS 2018 wave, 30 factors including individual characteristics, health behaviors, living environment, family economic status, and social participation, were analyzed in this study. To ensure the model’s reliability, the study cohort was randomly split into a training set (80%) and a validation set (20%). The *χ*^2^ tests and a Least Absolute Shrinkage and Selection Operator (LASSO) regression analysis were used to identify the most effective predictors of the model. The logistic regression model was employed to investigate the factors associated with depressive symptoms in perimenopausal women with somatic pain. A nomogram was constructed to develop a prediction model, and calibration curves were used to assess the accuracy of the nomogram model. The model’s performance was evaluated using the area under the curve (AUC) and decision curve analysis (DCA).

**Results:**

In total, 2,265 perimenopausal women were included in the final analysis, of whom 1,402 (61.90%) experienced somatic pain. Multifactorial logistic regression identified marital status, pain distress, self-perceived general health, activities of daily living (ADL), sleep deprivation, life satisfaction, and air quality satisfaction, as predictive risk factors for perimenopausal women with somatic pain. The predictive model achieved an AUC of 0.7010 (95%*CI* = 0.677–0.725) in the training set and 0.7015 (95%*CI* = 0.653–0.749) in the validation set. The nomogram showed excellent predictive ability according to receiver operating characteristic (ROC) and DCA, and the model may help in the early detection of high-risk depression symptoms in perimenopausal women with somatic pain, thereby enabling the timely initiation of appropriate treatment interventions.

**Conclusion:**

The nomogram constructed in this study can be used to identify the factors related to depression in women with perimenopausal somatic pain.

## 1 Introduction

Based on the Stages of Reproductive Aging Workshop (STRAW) findings, perimenopause is the period surrounding menopause (1). Women in the perimenopausal stage not only experience physiological changes due to fluctuations in sex hormones, but also exhibit a range of symptoms associated with menopause transition. They may experience irregular menses, vasomotor symptoms, sleep disruption, mood disorders as well as genitourinary symptoms. Additionally, they often face increased pressures in their family, social, and economic lives (2, 3), which can lead to mood changes and even clinical manifestations of depression (4).

Approximately 70% of depression cases in middle-aged women occur during the perimenopausal period (4), and severe depression has become the second leading cause of health impairment among women (5). Furthermore, there is a correlation between fluctuations in sex hormones and pain levels in women suffering from somatic pain. Clinical studies have shown that the levels of two sex hormones, estradiol and testosterone, are significantly correlated with pain levels (6, 7). This suggests that there may be shared changes in the brain regions and chemical substances involved in the pathophysiological mechanisms of both pain and depression (8).

Additionally, the pain threshold in patients with depression is significantly higher than in non-depressed individuals, and there is a notable positive correlation between the severity of depression and pain sensitivity (9, 10). Medical research has shown that there is a complex and strong association between somatic pain and depression in perimenopausal women. The significant fluctuation of estrogen levels due to the decline of ovarian function not only has a significant impact on the regulation of the nervous system, but may also further enhance the intrinsic association between chronic pain and depression through the neuroendocrine pathway. The sharp decline in estrogen levels during this period may lower the threshold of pain sensation in women, thus making them more sensitive to pain (11). This leads to a preliminary conclusion that there may be a coupling relationship between fluctuations in sex hormones, depressive symptoms, and pain levels in women. Given that women in the perimenopausal stage experience more pronounced fluctuations in sex hormones, they are at a higher risk for comorbid pain and depression.

While there is a growing body of research focusing on women with perimenopausal depression (12, 13) or somatic pain (14), there is currently a dearth of tools available for the prediction thereof. We, therefore, aimed to identify key modifiable factors influencing depression in perimenopausal women with somatic pain, to develop a clinically actionable nomogram for individualized risk stratification, which will provide a reference for early screening and thereby enabling the timely initiation of appropriate treatment interventions.

## 2 Materials and methods

The China Health and Retirement Longitudinal Study (CHARLS) aims to collect a high-quality nationally representative sample of Chinese residents ages 45 and older to serve the needs of scientific research on older adults, details of which are publicly available at http://charls.pku.edu.cn. The baseline survey began in 2011 and included 150 county-level units and 450 village-level units, with 17,008 participants. Follow-ups are conducted every 2 years, and to date, four waves of data are available. The project uses multistage probability proportional to size (PPS) sampling at the county/district and village/community levels.

Our study used the fourth wave of data (2018), which includes 19,816 participants covering all 30 provinces, autonomous regions, and municipalities directly under the central government, ensuring national representativeness (15). This study was based on secondary analyses of publicly anonymized data from the CHARLS, a large-scale longitudinal survey conducted by the China Social Science Research Center at Peking University, whose original research protocol was approved by the Ethics Committee of Peking University (Approval No. IRB00001052-11015), and strictly complied with the Declaration of Helsinki and the relevant norms of China’s Measures for Ethical Review of Biomedical Research Involving Human Beings. Based on the objectives of this study and existing research (16–18), the inclusion criteria for the study participants were as follows: females; aged between 45 and 60 years; and reporting at least one site of somatic pain (including the head, shoulders, arms, wrists, fingers, chest, back, waist, hips, legs, knees, ankles, toes, and neck). Samples were screened according to these criteria, and those with missing data on relevant variables were also excluded. A total of 2,265 perimenopausal women with somatic pain were selected for the study.

### 2.1 Dependent variables

The Center for Epidemiologic Studies Depression Scale (CESD-10) was used to assess depressive symptoms. The scale consists of eight items describing negative emotions and two describing positive ones. Response options are scored as follows: Rarely or none of the time (<1 day) = 0 points; Some or a little of the time (1–2 days) = 1 point; Occasionally or a moderate amount of the time (3–4 days) = 2 points; and most or all of the time (5–7 days) = 3 points. After reversing the scores for the two reverse-coded items, the total score was calculated to obtain the CESD-10 score for the sample. The total scores on the CESD-10 range from 0 to 30, with scores ≥10 defining depression and scores <10 indicating the absence of depression. The CESD-10 Scale indicated adequate reliability and validity for the community-dwelling older population in China, effectively measuring depression levels in middle-aged and older adults (19, 20).

### 2.2 Independent variables

Drawing on existing research (21, 22), our study initially selected 30 individual indicators as independent variables based on five dimensions: individual characteristics, health behaviors, living environment, family economic status, and social participation. The assignment methods for each relevant independent variable are shown in Table 1.

**Table 1:** Selected explanatory variables and assigned values.

Variable name	Assignment method	Variable name	Assignment method
Individual characteristics		Living environment	
Education level	Junior High School and Above = 1; Primary School and Below = 2; Illiterate = 3	Residence	Urban = 0; Rural = 1
Marital status	Married = 0; Unmarried = 1	Life satisfaction	Satisfied = 0; Unsatisfied = 1
Ethnicity	Han = 0; Ethnic Minorities = 1	Residential area	Eastern Region = 0; Midwestern Region = 1
Religious belief	No = 0; Yes = 1	Coastal city	No = 0; Yes = 1
Employment status	No = 0; Yes = 1	Air quality satisfaction	Satisfied = 0; Unsatisfied = 1
Vigorous-intensity activity	No = 0; Yes = 1	Living alone	No = 0; Yes = 1
Menstrual situation	No = 0; Yes = 1	Family economic status	
Self-perceived health status	Good = 1; Fair = 2; Poor = 3	Poor family	No = 0; Yes = 1
Pain distress	Rarely = 0; Often = 1	Family size	1 = 1; 2 = 2; 3 = 3; 4 = 4; > 4 = 5
Chronic diseases	No = 0; Yes = 1	Number of children	1 = 0; > 1 = 1
ADL	No = 0; Yes = 1	The burden of raising children	No = 0; Yes = 1
Health behaviors		Social participation	
Smoke	No = 0; Yes = 1	Social activities	Daily = 4; Weekly = 3; Infrequently = 2; None = 1
Drink	No = 0; Yes = 1	Internet usage	No = 0; Yes = 1
Napping habits	No = 0; Yes = 1	Insurance	uninsured = 0; insured = 1
Sleep deprivation	≥6 h = No = 0; < 6 h = Yes = 1	Pension	uninsured = 0; insured = 1

Age is a continuous numerical variable. Marital status was recorded as follows: (0 = married and living with spouse/ cohabitated; 1 = separated/divorced/never married/widowed). Respondents who reported participating in vigorous-intensity activities: carrying heavy items, digging, hoeing, aerobic workouts, bicycling at a fast speed, and riding a cargo bike/motorcycle, were recorded as follows: 1 = at least 5 days a week and more than 4 h each day; 0 = otherwise. The pain distress indicator is based on respondents’ answers to the question, “Are you often troubled with anybody pains?“0 points were recorded for responses indicating “a little” or “somewhat” (less frequent), whereas 1 point was recorded for responses indicating “quite a bit” or “very much” (more frequent). For the presence of chronic diseases, respondents were asked whether they have been diagnosed with any of 14 chronic conditions by a doctor, including hypertension, dyslipidemia, diabetes, cancer, lung disease, liver disease, heart disease, stroke, kidney disease, stomach disease, mental illness, memory-related diseases, arthritis or rheumatism, and asthma. If a respondent had at least one chronic disease, the answer was recorded as “1″; if no diseases were reported “0″.

In the CHARLS data, the activities of daily living (ADL) component were assessed in six aspects including dressing, bathing, eating, toilet, transfer, and controlling urination and defecation. Following relevant studies (23, 24), if a respondent reported difficulty or the inability to perform any activity, they were classified as ADL = and “1”; alternatively, this was recorded as otherwise, as “0”; The residential area was based on the province where the respondent resides. (Eastern Region: Beijing, Tianjin, Hebei, Shandong, Jiangsu, Shanghai, Zhejiang, Fujian, Guangdong, Hainan; Midwestern Region: Shanxi, Henan, Anhui, Hubei, Jiangxi, Hunan, Chongqing, Sichuan, Yunnan, Guizhou, Guangxi, Tibet, Shaanxi, Gansu, Ningxia, Qinghai, Xinjiang, Inner Mongolia, Liaoning, Jilin, Heilongjiang). Coastal cities were identified based on whether the respondent’s city was classified as a coastal city (1 = Yes, 0 = No). The poverty variable was defined according to the poverty standard set by the National Bureau of Statistics in 2018, which states that a household is considered poor if the per capita annual income or consumption is below 3,000 yuan (approximately 2,995 yuan). Households with a yearly consumption per capita below 3,000 yuan were classified as poor 1 point, while those above this threshold were classified as non-poor 0 points.

### 2.3 Statistical methods

The statistical software we used for the analysis was STATA /MP 17.0. Categorical variables were described using frequency and percentage, while continuous variables were described using median (P25–75). Comparisons between groups were performed using the *χ*^2^ test. Data were randomly divided into training (*n* = 1,814) and validation (*n* = 451) sets, according to a ratio of 8:2. Influencing factors were selected using the LASSO regression model, while multivariate analysis used the logistic regression model.

In the research process, we employed the *χ*^2^test to compare the baseline characteristics of depression status among training and validation sets to ensure the appropriateness of the groupings, followed by a univariate analysis of the 30 influencing factors in the training set. Next, the LASSO regression model was used to filter relevant characteristic variables. Subsequently, the selected variables were treated as independent variables, with the depressive symptoms of perimenopausal women with somatic pain serving as the dependent variable. A multivariate logistic regression model was constructed to identify the risk factors associated with depressive symptoms in this group.

Finally, based on the identified risk factors for depressive symptoms, a nomogram prediction model for depression risk in perimenopausal women with somatic pain was developed. The model’s predictive value was assessed using the area under the receiver operating characteristic (ROC) curve, and the calibration curve was used to evaluate the model’s fit. Additionally, DCA was employed to assess the clinical value of the nomogram prediction model. The study sets the statistical significance level was set at *α* = 0.05, meaning that *p* < 0.05 was considered statistically significant for group differences.

## 3 Results

### 3.1 Basic characteristics

Among the 2,265 women with somatic pain, 1,558 (68.79%) were postmenopausal, while 707 (31.21%) were perimenopausal. The average score on the CES-D10 Scale for all participants was 11.80 ± 4.96. In total, 863 women (31.21%) had a CES-D10 score of less than 10, indicating no depressive symptoms, whereas 1,402 women (61.90%) had a CES-D10 score of 10 or higher, indicating the presence of depressive symptoms. Comparisons between the training and validation sets are shown in Table 2, with no significant differences between the two groups (*p* > 0.05).

**Table 2:** Baseline characteristics of the study population.

Variable	Overall (*n* = 2,265)	training set (*n* = 1814)	validation set (*n* = 451)	*p*-value
Age	53.07 (50, 56)	53.1 (50, 56)	52.94 (50, 56)	0.406
Education (%)				0.58
Junior high school and above	801 (35.4)	107 (5.9)	168 (37.3)	
Elementary school or below	536 (23.6)	429 (23.7)	107 (23.7)	
Illiterate	928 (41)	752 (41.3)	176 (39)	
Marital (%)				0.846
Married	2,109 (93.1)	1,690 (93.2)	419 (92.9)	
Unmarried	156 (6.9)	124 (6.8)	32 (7.1)	
Ethnicity (%)				0.367
Han	2033 (89.8)	1,623 (89.5)	410 (90.9)	
Ethnic minorities	232 (10.2)	191 (10.5)	41 (9.1)	
Religious belief (%)				0.522
Yes	252 (11.1)	198 (10.9)	54 (12.0)	
No	2013 (88.9)	1,616 (89.1)	397 (88.0)	
Self-perceived health status (%)				0.472
Good	683 (30.2)	555 (30.6)	128 (28.4)	
Fair	1,221 (53.9)	967 (53.3)	254 (56.3)	
Poor	361 (15.9)	292 (16.1)	69 (15.3)	
Employment Status (%)				0.762
Yes	1,680 (74.2)	1,348 (74.3)	332 (73.6)	
No	585 (25.8)	466 (25.7)	119 (26.4)	
Vigorous-intensity activity (%)				0.237
Yes	520 (23)	407 (22.4)	113 (25.1)	
No	1745 (77)	1,407 (77.6)	338 (74.9)	
Menstrual situation (%)				0.98
Yes	1,558 (68.8)	1,248 (68.8)	310 (68.7)	
No	707 (31.2)	566 (31.2)	141 (31.3)	
Pain distress (%)				0.092
Often	640 (28.3)	527 (29.1)	113 (25.1)	
Rarely	1,625 (71.7)	1,287 (70.9)	338 (74.9)	
Chronic diseases (%)				0.109
Yes	1,091 (48.2)	889 (49)	202 (44.8)	
No	1,174 (51.8)	925 (51)	249 (55.2)	
ADL				0.497
Yes	386 (17)	314 (17.3)	72 (16)	
No	1879 (83)	1,500 (82.7)	379 (84)	
Smoke				0.859
Yes	97 (4.3)	77 (4.2)	20 (4.4)	
No	2,168 (95.7)	1737 (95.8)	431 (95.6)	
Drink				0.653
Yes	356 (15.7)	282 (15.5)	74 (16.4)	
No	1909 (84.3)	1,532 (84.5)	377 (83.6)	
Napping habit				0.891
Yes	1,244(54.9)	995(54.8)	249(55.3)	
No	1,021(45.1)	819(45.2)	202(44.7)	
Sleep deprivation				0.133
Yes	919 (40.6)	722 (39.8)	197 (43.7)	
No	1,346 (59.4)	1,092 (60.2)	254 (56.3)	
Residence				0.875
Urban	561 (24.8)	448 (24.7)	113 (25.1)	
Rural	1,704 (75.2)	1,366 (75.3)	338 (74.9)	
Life Satisfaction				0.647
Satisfied	1,882 (83.1)	1,504 (82.9)	378 (83.8)	
Unsatisfied	383 (16.9)	310 (17.1)	73 (16.2)	
Residential area				0.458
Eastern	562 (24.8)	444 (24.5)	118 (26.2)	
Midwestern	1,703 (75.2)	1,370 (75.5)	333 (73.8)	
Coastal city				0.957
Yes	293 (12.9)	235 (12.9)	58 (12.9)	
No	1,972 (87.1)	1,579 (87.1)	393 (87.1)	
Air quality satisfaction				0.21
Satisfied	1,801 (79.5)	1,452 (80.1)	349 (77.4)	
Unsatisfied	464 (20.5)	362 (19.9)	102 (22.6)	
Living alone				0.929
Yes	87 (3.8)	70 (3.8)	17 (3.8)	
No	2,178 (96.2)	1,744 (96.2)	434 (96.2)	
Poor family				0.378
Yes	103 (4.5)	79 (4.4)	24 (5.3)	
No	2,162 (95.5)	1,735 (95.6)	427 (94.7)	
Family size				0.433
1	87 (3.8)	70 (3.9)	17 (3.8)	
2	1,025 (45.3)	827 (45.6)	198 (43.9)	
3	577 (25.5)	463 (25.5)	114 (25.2)	
4	282 (12.4)	214 (11.8)	68 (15.1)	
>4	294 (13.0)	240 (13.2)	54 (12.0)	
Number of children				0.854
1	560 (24.7)	450 (24.8)	110 (24.4)	
>1	1,705 (75.3)	1,364 (75.2)	341 (75.6)	
The burden of raising children				0.377
Yes	1,128 (49.8)	895 (49.3)	233 (51.7)	
No	1,137 (50.2)	919 (50.7)	218 (48.3)	
Social activities				0.609
None	925 (40.9)	740 (40.8)	185 (41.1)	
Infrequently	358 (15.8)	293 (16.2)	65 (14.4)	
Weekly	229 (10.1)	177 (9.7)	52 (11.5)	
Daily	753 (33.2)	604 (33.3)	149 (33.0)	
Internet usage				0.164
Yes	398 (17.6)	325 (17.9)	73 (16.2)	
No	1,867 (82.4)	1,489 (82.1)	378 (83.8)	
Insurance				0.953
Insured	2,219 (98)	1,777 (98)	442 (98)	
Uninsured	46 (2)	37 (2)	9 (2)	
Pension				0.483
Insured	328 (14.5)	258 (14.2)	70 (15.5)	
Uninsured	1,937 (85.5)	1,556 (85.8)	381 (84.5)	

When comparing these findings with existing research, it was observed that the detection rate of depressive symptoms among perimenopausal women with somatic pain was significantly higher than in rural middle-aged and older women aged over 45 years (42.46%) (4) and the detection rate of depressive symptoms in all perimenopausal women (35.5%) (19).

### 3.2 Univariate analysis of depressive symptoms

At the individual characteristic level, the comparison of detection rates of depressive symptoms among perimenopausal women with somatic pain revealed statistically significant differences among groups based on education level, marital status, self-perceived general health status, pain distress, chronic diseases, and ADL (*p* < 0.05). At the level of health behaviors, however, there was a statistically significant difference in the detection rates of depressive symptoms among perimenopausal women with somatic pain based on different sleep durations (*p* < 0.05). At the level of the living environment, comparisons of detection rates of depressive symptoms among perimenopausal women with somatic pain based on whether they live in rural areas, overall life satisfaction, residential area, whether they reside in coastal cities, air quality satisfaction, and living alone showed statistically significant differences among the groups (*p* < 0.05). At the level of family economics, the comparison of detection rates of depressive symptoms among perimenopausal women with somatic pain based on the number of children and the burden of raising children showed statistically significant differences among the groups (*p* < 0.05). At the level of social participation, comparisons of detection rates of depressive symptoms among perimenopausal women with somatic pain showed statistically significant differences among the groups only based on the status of pension insurance enrollment (*p* < 0.05).

In summary, the differences among groups in 16 characteristic variables, including education level, marital status, self-perceived general health status, pain distress, chronic diseases, ADL, sleep deprivation, residence, life satisfaction, residential area, coastal city status, air quality satisfaction, living alone, number of children, the burden of raising children, and pension insurance enrollment, are statistically significant. The results are presented in Table 3.

**Table 3:** Univariate analysis of depressive symptoms in a training set of perimenopausal women experiencing somatic pain.

Variable	Depression (*n* = 1,118)	Non-depression (*n* = 696)	*p*
Age	53.26 (50, 56)	52.84 (49, 56)	0.292
Education (%)			0.001
Junior high school and above	355 (31.8)	278 (39.9)	
Elementary school or below	267 (23.8)	162 (23.3)	
Illiterate	496 (44.4)	256 (36.8)	
Marital (%)			0.001
Married	1,024 (91.6)	666 (95.7)	
Unmarried	94 (8.4)	30 (4.3)	
Ethnicity (%)			0.291
Han	1,007 (90.1)	616 (88.5)	
Ethnic minorities	111 (9.9)	80 (11.5)	
Religious belief (%)			0.519
Yes	991 (88.6)	625 (89.8)	
No	127 (11.4)	71 (10.2)	
Self-perceived health status (%)			0
Good	415 (37.1)	140 (20.1)	
Fair	572 (51.2)	395 (56.8)	
Poor	131 (11.7)	161 (23.1)	
Employment Status (%)			0.453
Yes	824 (73.7)	524 (75.3)	
No	294 (26.3)	172 (24.7)	
Vigorous-intensity activity (%)			0.55
Yes	256 (22.9)	151 (21.7)	
No	862 (77.1)	545 (78.3)	
Menstrual situation (%)			0.079
Yes	786(70.3)	462(66.4)	
No	332(29.7)	234(33.6)	
Pain distress (%)			0
Often	395 (35.3)	132 (19)	
Rarely	723 (64.7)	564 (81)	
Chronic diseases (%)			0.005
Yes	577 (51.6)	312 (44.8)	
No	541 (48.4)	384 (55.2)	
ADL			0
Yes	240 (21.5)	74 (10.7)	
No	878 (78.5)	622 (89.3)	
Smoke			0.727
Yes	46 (4.1)	31 (4.5)	
No	1,072 (95.9)	665 (95.5)	
Drink			0.337
Yes	181 (16.2)	101 (14.5)	
No	937 (83.8)	595 (85.5)	
Napping habit			0.115
Yes	597 (53.4)	398 (57.2)	
No	521 (46.6)	298 (42.8)	
Sleep deprivation			0
Yes	529 (47.3)	193 (27.7)	
No	589 (52.7)	503 (72.3)	
Residence			0.032
Urban	257 (23)	191 (27.4)	
Rural	861 (77)	505 (72.6)	
Life Satisfaction			0
Satisfied	263 (23.5)	47 (6.8)	
Unsatisfied	855 (76.5)	649 (93.2)	
Residential area			0.002
Eastern	246 (22)	198 (28.0)	
Midwestern	872 (78)	498 (72.0)	
Coastal city			0.003
Yes	124 (11)	111 (16)	
No	994 (89)	585 (84)	
Air quality satisfaction			0
Satisfied	254 (22.7)	108 (15.5)	
Unsatisfied	864 (77.3)	588 (84.5)	
Living Alone			0.049
Yes	51 (4.6)	19 (2.7)	
No	1,067 (95.4)	677 (97.3)	
Poor family			0.084
Yes	56 (5)	23 (3.3)	
No	1,062 (95)	673 (96.7)	
Family size			0.164
1	51 (4.6)	19 (2.8)	
2	522 (46.7)	305 (43.8)	
3	277 (24.8)	186 (26.7)	
4	125 (11.2)	89 (12.8)	
>4	143 (12.7)	97 (13.9)	
Number of children			0.002
1	250 (22.4)	200 (28.7)	
>1	868 (77.6)	496 (71.3)	
The burden of raising children			0.029
Yes	529 (47.3)	366 (52.6)	
No	589 (52.7)	330 (47.4)	
Social activities			0.407
None	444 (39.7)	296 (42.5)	
Infrequently	184 (16.4)	109 (15.7)	
Weekly	118 (10.6)	59 (8.5)	
Daily	372 (33.3)	232 (33.3)	
Internet Usage			0.241
Yes	191 (17.1)	134 (19.2)	
No	927 (82.9)	562 (80.8)	
Insurance			0.683
Insured	1,094 (97.8)	683 (98.1)	
Uninsured	24 (2.1)	13 (1.9)	
Pension			0.006
Insured	139 (12.4)	119 (17.1)	
Uninsured	979 (87.6)	577 (82.9)	

### 3.3 Influencing factors based on the LASSO regression model

**Figure 1:**
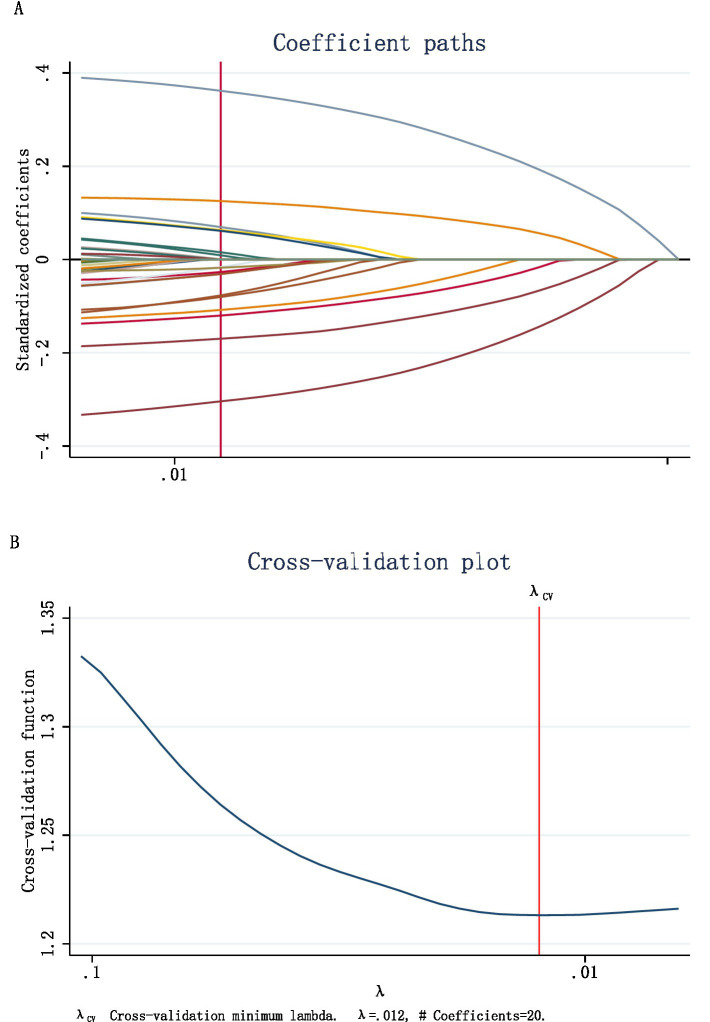
**(A)** According to the logarithmic (lambda) sequence, a coefficient profile was generated, and non-zero coefficients were produced by the optimal lambda. **(B)** The optimal parameter (lambda) in the LASSO model was selected via tenfold cross-validation using minimum criteria.

The 30 influencing factors related to depressive symptoms in perimenopausal women with somatic pain were incorporated into a LASSO regression model. A 10-fold cross-validation method was used to select the optimal *λ*, which was then applied to further filter the influencing factors. The results are shown in Figures 1A,B.

It can be observed that the LASSO regression model included 20 dummy variables when the optimal λ value was set at 0.012. After further organizing and consolidating these dummy variables, 17 characteristic factors influencing depressive symptoms in perimenopausal women with somatic pain were identified. These factors include individual characteristics, such as age, ethnicity, education level, marital status, pain distress, self-perceived general health, and ADL; Health behaviors: drinking habit, and sleep deprivation. Living environment: residential area, coastal city, life satisfaction, and air quality satisfaction; family economic factors: number of children and the burden of raising children; social participation: frequency of social interactions, and pension insurance enrollment.

### 3.4 Multivariate logistic regression analysis

First, we identified the intersection of the 17 variables selected based on the LASSO regression model and the 16 variables that showed statistically significant differences between groups. This process resulted in 13 characteristic variables related to depressive symptoms in perimenopausal women with somatic pain. These variables include education level, marital status, pain distress, self-perceived general health, ADL, sleep deprivation, life satisfaction, air quality satisfaction, residential area, coastal city status, number of children, the burden of raising children, and pension enrollment.

Considering the solid logical correlation between the variables of residential area and coastal city status, we excluded the variables of residential area to avoid serious multicollinearity issues in the model. The remaining 12 factors were included as independent variables, with depressive symptoms in perimenopausal women with somatic pain as the dependent variable.

The analysis indicated that unmarried status, higher levels of pain distress, poor self-perceived health, ADL, sleep deprivation, low life satisfaction, and low air quality satisfaction are risk factors for depressive symptoms in perimenopausal women with somatic pain. A multivariate logistic regression model was constructed for analysis, and the results are presented in Table 4.

**Table 4:** Logistic regression of factors influencing depressive symptoms in perimenopausal women with somatic pain.

Variable	*β*	*SE*	*p*	*OR*	95%*CI*
Marital status (Married)					
Non-Married	0.580	0.23	0.011	1.78	(1.14, 2.79)
Pain distress (Rarely)					
Often	0.485	0.13	<0.001	1.62	(1.27, 2.08)
Self-perceived health status (Good)					
Fair	−0.315	0.13	0.015	0.73	(0.57, 0.94)
Poor	−0.711	0.17	<0.001	0.49	(0.35, 0.68)
ADL (No)					
Yes	0.444	0.16	0.004	1.56	(1.15, 2.16)
Sleep deprivation (No)					
Yes	0.739	0.11	<0.001	2.09	(1.69, 2.59)
Life Satisfaction (Satisfied)					
Unsatisfied	−1.122	0.18	<0.001	0.33	(0.23, 0.46)
Air quality satisfaction (Satisfied)					
Unsatisfied	−0.289	0.14	0.034	0.75	(0.57, 0.98)
Constant term	1.470	0.21	<0.001	0.94	

### 3.5 Construction and validation of the nomogram model

**Figure 2:**
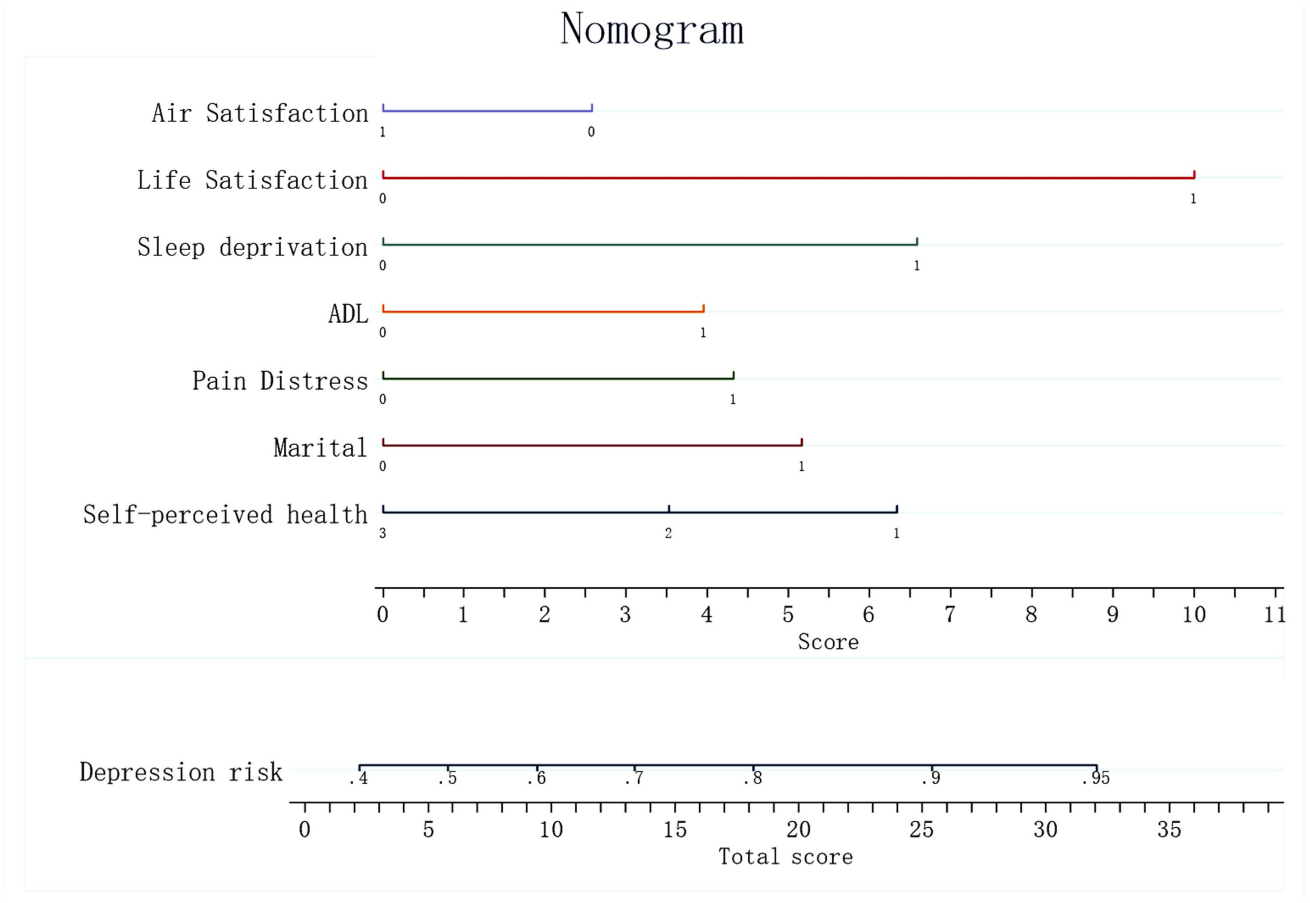
Nomogram.

Based on the significant characteristic factors identified in the logistic regression model that influence the probability of depression in perimenopausal women with somatic pain—marital status, pain distress, self-perceived general health, ADL, sleep deprivation, life satisfaction, and air quality satisfaction—a nomogram prediction model was constructed (Figure 2). A nomogram is a visual tool used to estimate the probability of depressive symptoms in this population by integrating the identified risk factors. Each factor is assigned a specific score based on its contribution to the overall risk, and the total score can be used to predict the likelihood of developing depressive symptoms.

**Figure 3:**
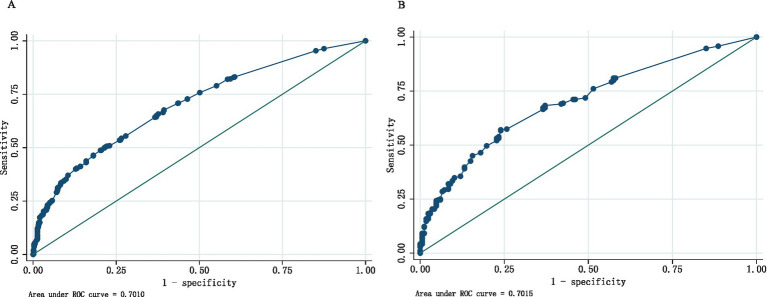
**(A)** ROC curves generated from the training data set. **(B)** ROC curves generated using the validation data set.

The detection of depressive symptoms in women with perimenopausal somatic pain was examined in both the training and validation sets, and the area under the curve (AUC) was calculated to assess the discriminative ability of the model, as shown in Figures 3A,B. The model had an AUC of 0.7010 in the training set, which is greater than the critical value of 0.7, indicating good predictive performance. In the validation set, the AUC was 0.7015. These results indicate that the nomogram has good discriminatory and predictive value in correctly identifying depressive symptoms and non-depressive symptoms.

**Figure 4:**
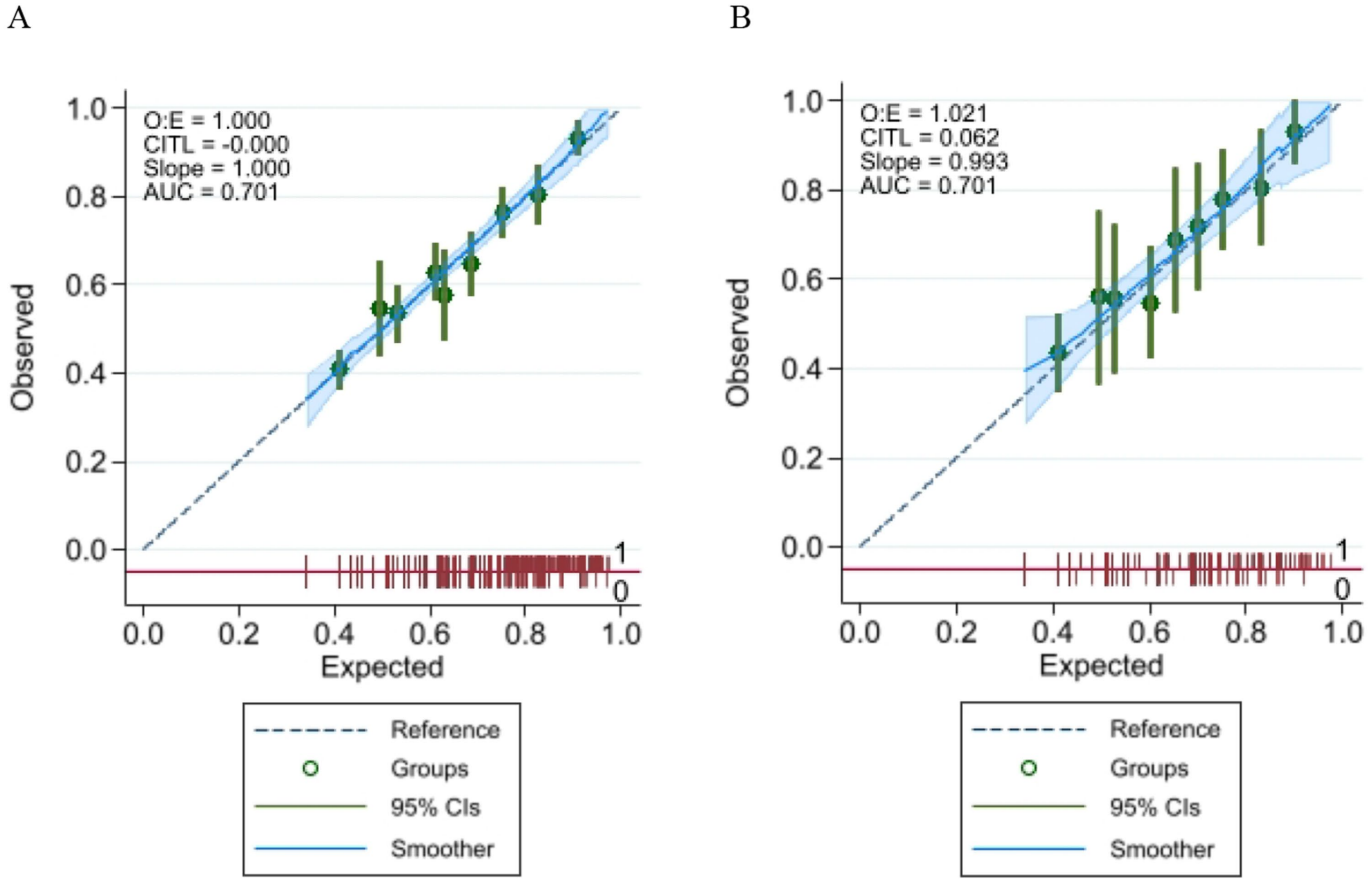
**(A)** Calibration plot for the training data set; **(B)** Calibration plot for the validation data set.

**Figure 5:**
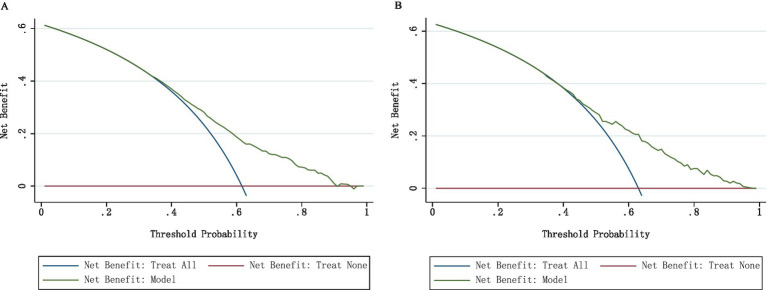
**(A)** DCA curves for the training data set. **(B)** DCA curves for the validation data set.

The nomogram was evaluated using calibration plots and the Hosmer-Lemeshow goodness-of-fit test (*p* > 0.05 indicates very good fit). The test results showed a good fit for both the training and validation sets. The test results showed that the model had a good fit for the training set (*χ*^2^ = 5.36, df = 7, *p* = 0.8021) and the validation set (*χ*^2^ = 2.73, df = 7, *p* = 0.9240). By plotting the calibration curve, we examined the consistency of the nomogram model for the training and validation sets shown in Figures 4A,B. The clinical validity of the model was evaluated using the DCA method, and the results are shown in Figures 5A,B.

## 4 Discussion

This study aimed to identify factors related to depression in women with perimenopausal somatic pain and incorporate them into the construction of a predictive model, which will provide a reference for early screening and thereby enabling the timely initiation of appropriate treatment interventions. Our successfully constructed model can be used to identify the factors related to depression in women with perimenopausal somatic pain. To our knowledge, this is the first study to integrate machine learning-derived predictors with traditional logistic models in this population, potentially bridging the gap between epidemiological research and precision prevention strategies.

Depressive symptoms significantly increase the disease burden in patients with somatic pain, and many chronic pain conditions are influenced not only by hormonal fluctuations but also by psychosocial changes (25). This makes them more prone to developing depressive symptoms (26). This study specifically addresses the issue of depression in perimenopausal women with somatic pain, and the results indicate that the depression detection rate among perimenopausal women with somatic pain in China is 61.90%. This level is significantly higher than the depression detection rate of 35.5% in the overall sample of perimenopausal women in China (4) and 43.61% in women aged 45 and older in China (27).

To this end, we identified and examined the risk factors for depressive symptoms in perimenopausal women with somatic pain from five dimensions: individual characteristics, health behaviors, living environment, family economy, and social participation. A nomogram prediction model was constructed to guide clinical management and decision-making.

We identified seven independent risk factors for depressive symptoms in perimenopausal women with somatic pain, including unmarried status, severe pain distress, poor self-reported health, and ADL. These findings align with prior research emphasizing the bidirectional relationship between pain and depression (28). The possible reasons include marital changes, declining health, and ADL which are adverse events that affect patients’ quality of life and well-being. Marriage can provide stability and emotional and psychological support for older adults facing major life changes (29), which may account for the association of perimenopausal somatic pain with depressive symptoms in unmarried women. These individual characteristics play a significant part in shaping negative emotions, influencing perceived social support, sense of control, and self-esteem. Researchers have determined that ADL can increase the risk of depression for middle-aged and older adults and their spouses (30). The findings suggest that initial levels of ADLs and instrumental IADLs are associated with the risk of developing depression, with higher initial levels associated with a higher risk thereof depression (31). Additionally, perimenopausal women experience estrogen fluctuations, which can have an impact on the manifestation of depressive symptoms. Hence, healthcare providers should give special consideration to this demographic.

Furthermore, the current results found that perimenopausal women with somatic pain who experience significant pain distress have a higher probability of depressive symptoms compared to those with less pain distress, indicating that considerable pain distress is a risk factor for depression in this population. This is also consistent with previous studies (32). Physiologically, the comorbidity of pain and depression may be driven by shared neuroendocrine pathways. Fluctuating estrogen levels during perimenopause reduce serotonin synthesis, lowering pain thresholds and exacerbating emotional dysregulation (33). Concurrently, chronic pain activates brain regions associated with affective processing (e.g., ACC and insula), creating a vicious cycle (8). These mechanisms highlight the need for integrated interventions targeting both pain and mood disorders. Currently, menopausal hormone therapy (HT) has shown promising results in addressing the comorbidity of somatic pain and depressive symptoms; however, hormone therapy is contraindicated in some patients due to cancer risk, and therefore other treatment options are necessary (34–36).

In terms of health behaviors, our findings explored the impact of smoking history, drinking habits, napping habits, and sleep duration on the detection of depression in perimenopausal women with somatic pain based on existing research findings. Notably, our LASSO-logistic model excluded variables such as smoking and alcohol use, contrasting with general population findings (37–39). This discrepancy may stem from the overriding impact of somatic pain in this specific cohort, overshadowing the effects of health behaviors. A possible reason for this is that although the prevalence of smoking habits is relatively low among perimenopausal women, they are often exposed to secondhand smoke in their family and social environments (40), which may explain the lack of a significant correlation between smoking history and the incidence of depression. Lastly, regarding sleep habits, there is no statistically significant difference in the depression incidence rate between groups with and without napping habits among perimenopausal women with somatic pain. However, sleep deprivation is identified as a risk factor for depression in this population. This finding may suggest that the quality of nighttime sleep, rather than total sleep duration, significantly influences depressive symptoms in perimenopausal women with somatic pain. Studies have linked worsening sleep quality to higher depression risk. Sleep quality may indirectly affect depression through pain and functional disability. For people with moderate levels of sleep quality, improving sleep quality may reduce the risk of depression (41, 42). Regarding health behaviors, people who exercise moderately and maintain a healthy diet have a lower risk of developing depressive symptoms compared to those with an unhealthy diet and low levels of exercise (43).

In terms of family economic status and social participation, this study explored the impact of factors such as family poverty, population size, number of children, caregiving burden, frequency of social interactions, internet usage, medical insurance coverage, and pension coverage on the probability of depression in perimenopausal women with somatic pain. The results indicated that although there were statistically significant differences in the number of children and pension insurance coverage between groups (16, 20, 26, 44), further LASSO-logistic regression results showed only low overall life satisfaction and low air quality satisfaction as risk factors for depressive symptoms in perimenopausal women with somatic pain. Air quality includes environmental factors such as air pollution, noise, heavy metals, and pesticides all affecting satisfaction with the air. These factors may increase the risk of depressive symptoms through biological mechanisms such as oxidative stress, neuroinflammation, and hormonal imbalance (45). A study of 75 cities in China found that short-term elevated concentrations of air pollutants increased the risk of hospitalization for depression in the population (46). Studies have shown that life satisfaction and depressive symptoms map to each other, with cognitive functioning acting as a mediating effect, positively predicting life satisfaction and negatively predicting depressive symptoms (47). People with depressive symptoms are more likely to exhibit negative attitudes toward life, and those who are dissatisfied with their lives are more likely to be at mental health risk (48).

We explored the effects of family poverty, population size, number of children, dependency burden, frequency of socialization, internet use, health insurance participation, and pension insurance participation on the probability of depression in perimenopausal women with somatic pain. The results showed that while the between-group differences in the number of children and pension insurance participation were statistically significant, the further LASSO-logistic regression results showed that neither was a risk factor for depressive symptoms in perimenopausal females with somatic pain. This result suggests that family economic and social participation factors are not major contributors to depression in these females.

The risk factors for depression symptoms in perimenopausal women with somatic pain include unmarried, significant pain distress, poor self-perceived general health, ADL, sleep deprivation, low life satisfaction, and low air quality satisfaction. A nomogram model that can predict symptoms of depression in perimenopausal women experiencing somatic pain was successfully developed and validated in this study. There should be more emphasis on the early detection and treatment of depression symptoms given the high prevalence of these symptoms in perimenopausal women experiencing somatic pain. Practice in the clinic needs to focus on risk factors that are weighted more heavily in the model (unmarried, ADL, significant pain distress, and sleep deprivation). Based on the predictive function of risk models, healthcare teams should strengthen early screening systems for such populations, especially by synchronizing mood assessment at the time of initial diagnosis of somatic pain, and dynamic monitoring for precise intervention of depressive symptoms.

It is noteworthy that Traditional Chinese medicine (TCM) theories provide a unique diagnostic and therapeutic perspective for such psychosomatic co-morbidities (49, 50). From the perspective of Chinese medicine, the comorbidity of depression and somatic pain includes manifestations such as pain and tendon inflexibility, characteristic of the “Bi syndrome” as well as symptoms of emotional distress. The role of the liver is to regulate mood and is closely related to the muscles of the body, thus regulating emotions. For instance, acupuncture has demonstrated efficacy in reducing depressive symptoms by modulating hypothalamic–pituitary–adrenal axis hyperactivity (18). Therefore, TCM interventions may be considered as a complementary option for perimenopausal women with depressive symptoms in regions where cultural acceptance and quality control of TCM are established.

Although the model demonstrated predictive ability, a significant limitation is that, as in most large population-based studies, the data for depression symptoms and other somatic pain were self-reported, and the data for our research comes from 6 years ago, and further updates of the latest data will be needed in the future to better analyze the results. Other limitations should be acknowledged. First, the cross-sectional nature of the study data may limit the universality and extrapolation of the results, which will require future validation in a multicenter setting and among perimenopausal women in different regions. Second, our study’s cross-sectional design inherently limits the ability to determine causal relationships between somatic pain and depression symptoms. Therefore, high-quality prospective studies should be conducted to verify this causal relationship. Thirdly, several potential risk factors, such as reduced physical activity and other medical histories, may also be at the root of depression symptoms, but are not included as covariates in our study. Fourth, we were unable to account for hormone therapy (HT) status as a potential confounding variable. The original dataset lacked detailed documentation on HT use, which may influence the association between somatic pain and depressive symptoms, given the known interactions between hormonal fluctuations and perimenopausal health status. Because our study was conducted only on women with perimenopausal somatic pain, the identified influences need to be interpreted with caution for other populations and systematically collect hormonal therapy data to address this gap. Therefore, future research should include more centers with a prospective design to increase the evidence and reliability of the results, which could be examined in a future study.

## 5 Conclusion

We developed a predictive model to assess factors influencing depressive symptoms in women with perimenopausal somatic pain. The model considers various factors including age, gender, place of residence, education level, pension, insurance, daily physical functioning, self-reported health status, and satisfaction with life. By utilizing the results of the assessment, early intervention can be implemented for the group of perimenopausal women with somatic pain, to improve the likelihood of reducing the occurrence of depressive symptoms.

## Data Availability

The original contributions presented in the study are included in the article/supplementary material, further inquiries can be directed to the corresponding authors.
